# EstuarySAT Database Development of Harmonized Remote Sensing and Water Quality Data for Tidal and Estuarine Systems

**DOI:** 10.3390/w16192721

**Published:** 2024-09-25

**Authors:** Steven A. Rego, Naomi E. Detenbeck, Xiao Shen

**Affiliations:** 1 Office of Research and Development, U.S. Environmental Protection Agency, Narragansett, RI 02882, USA;; 2 College of Engineering, Computing and Cybernetics, The Australian National University, Canberra, ACT 2601, Australia;

**Keywords:** remote sensing, water quality, chlorophyll, Sentinel-2, harmful algal blooms

## Abstract

Researchers and environmental managers need big datasets spanning long time periods to accurately assess current and historical water quality conditions in fresh and estuarine waters. Using remote sensing data, we can survey many water bodies simultaneously and evaluate water quality conditions with greater frequency. The combination of existing and historical water quality data with remote sensing imagery into a unified database allows researchers to improve remote sensing algorithms and improves understanding of mechanisms causing blooms. We report on the development of a water quality database “EstuarySAT” which combines data from the Sentinel-2 multi-spectral instrument (MSI) remote sensing platform and water quality data throughout the coastal USA. EstuarySAT builds upon an existing database and set of methods developed by the creators of AquaSat, whose region of interest is primarily larger freshwater lakes in the USA. Following the same basic methods, EstuarySAT utilizes open-source tools: R v. 3.24+ (statistical software), Python (dynamic programming environment), and Google Earth Engine (GEE) to develop a combined water quality data and remote sensing imagery database (EstuarySAT) for smaller coastal estuarine and freshwater tidal riverine systems. EstuarySAT fills a data gap that exists between freshwater and estuarine water bodies. We are able to evaluate smaller systems due to the higher spatial resolution of Sentinel-2 (10 m pixel image resolution) vs. the Landsat platform used by AquaSat (30 m pixel resolution). Sentinel-2 also has a more frequent revisit (overpass) schedule of every 5 to 10 days vs. Landsat 7 which is every 17 days. EstuarySAT incorporates publicly available water quality data from 23 individual water quality data sources spanning 1984–2021 and spatially matches them with Sentinel-2 imagery from 2015–2021. EstuarySAT currently contains 299,851 matched observations distributed across the coastal USA. EstuarySAT’s primary focus is on collecting chlorophyll data; however, it also contains other ancillary water quality data, including temperature, salinity, pH, dissolved oxygen, dissolved organic carbon, and turbidity (where available). As compared to other ocean color databases used for developing predictive chlorophyll algorithms, this coastal database contains spectral profiles more typical of CDOM-dominated systems. This database can assist researchers and managers in evaluating algal bloom causes and predicting the occurrence of future blooms.

## 1. Introduction

In recent decades, increases in eutrophic conditions in coastal systems have led to an increased frequency of harmful algal blooms (HAB) [1–6]. HABs can cause wide-ranging effects on the ecology of systems, local economies, commercial fisheries, and human health. Commercial fisheries are often hardest hit due to shellfish bed closures and mortality effects on fishery populations [7,8]. These effects are the result of the production of toxic species of phytoplankton and cyanobacteria or of secondary effects such as hypoxia. In 2018, the National Marine Fishery Service reported that HAB-related impacts on commercial fisheries totaled over 5.6 billion USD [8].

The causes of HAB events are being actively researched and may be influenced by the confluence of many environmental factors (biotic and abiotic). It is known that sustained increases in nitrogen and phosphorus inputs have played a role as primary drivers of increased HAB events and their duration [7,9,10]. Climate change has also contributed to these conditions adding to changes in mean water temperatures, salinity, rainfall, and sea-level rise that may also contribute to HAB frequency and duration [11].

Increased awareness by the public and reported health-related effects on humans and animals have resulted in increased monitoring of HABs and the water quality indicators that may contribute to these events. Developing databases to assess trends in HABs and associated conditions can be both time consuming and expensive [12]. There are many ongoing monitoring programs focused on the monitoring and prediction of HAB events in freshwater systems including those that supply drinking water [13]. Similarly, in large coastal estuaries such as the Chesapeake Bay, there are robust water quality monitoring programs. These programs are developing large-scale open-source water quality datasets that are maintained by various state, federal, and research institutions such as the Water Quality Portal (United States Geological Survey, USGS) and AquaSat [14,15] (Table 1). However, there are few monitoring programs or databases developed for HABs in tidal riverine and small coastal systems (e.g., https://mywaterquality.ca.gov/habs/, accessed on 8 August 2021).

An alternative approach to HAB field monitoring is the use of high-resolution remote sensing imagery to collect multispectral data across a wide area in a single satellite pass. Multispectral satellite sensors can detect changes in water quality by collecting surface reflectance containing the spectral characteristics of the water column. In open oceans, remote sensing technology has been used for over 50 years and has led to the production of large publicly available datasets combining in situ and remote sensing reflectance over long time periods [16]. Many algorithms have been developed from these datasets to estimate chlorophyll concentrations [17]. Early satellite platforms, such as Coastal Zone Color Scanner (CZCS—launched in 1978), Moderate Resolution Imaging Spectroradiometer (MODIS), and Medium Resolution Imaging Spectrometer (MERIS), had the ability to survey large swaths of open ocean but had poor spatial resolution. Although some of these platforms had high-frequency revisit times (e.g., 2-day revisit period for MODIS), their use for small-scale system research was limited due to their poor spatial resolution and, thus, inability to resolve smaller coastal estuaries and tributaries [18].

Today, state-of-the-art satellite platforms have been launched and are providing improved data at higher spatial resolutions and a broader range of wavelengths. Platforms such as the European Space Agency’s Sentinel-2 multi-spectral imaging system and NASA’s Landsat 7 system have a higher spatial resolution (10 and 30 m, respectively) and more frequent revisit times than earlier platforms. Sentinel-2 comprises a dual satellite system, Sentinel-2A and 2B, each being identical in spatial, temporal, and spectral resolutions. With two operational platforms in orbit, the Sentinel-2 revisit time is only 5 days compared with Landsat 7, which has a revisit time of 16 days. For these reasons, we chose to use Sentinel-2 imagery data for this project.

Satellite imagery can be combined with field and in situ water quality data to develop predictive algorithms to fill in spatial and temporal gaps in field monitoring. Previously, combined databases such as those provided by AquaSat included primarily freshwater data and only limited estuarine data [15]. AquaSat combines data from Landsat 5, 7, and 8 with data from the National Water Quality Data Portal (NWQP) [14]. AquaSat consists of a database of matched remote sensing and water quality constituents. The AquaSat database contains over 600,000 matching data points over a temporal period spanning 1984–2019. Parameters incorporated in this database include total suspended solids (TSS), dissolved organic carbon (DOC), water color, chlorophyll a, and Secchi depth (SD). The developers of AquaSat also provide open-source tools and scripts to allow others to utilize their methods.

The primary goal of our research is to develop a database containing a set of geospatially matched water quality data and remote sensing imagery for given time periods that includes chlorophyll and other ancillary water quality parameters (where available) for small estuaries and tidal rivers of the coterminous United States. This database will provide researchers and environmental managers with matched time-series data of imagery and water quality enabling them to develop new algorithms for chlorophyll estimation and HAB conditions across a range of systems with different optical characteristics.

Although there are many examples of the successful application of remote sensing for chlorophyll mapping in individual estuaries around the globe, most of these are based on limited calibration datasets [19–22], and more robust testing is needed of combinations of chlorophyll algorithms and processors for atmospheric correction [19,23]. More robust testing has been conducted for the processing of Sentinel-2 and Sentinel-3 images to predict chlorophyll in lakes [24], but the effectiveness of some algorithms can vary among optical water types (OWTs, [20,24,25]). Historically, most researchers have distinguished between Class I waters, which are dominated by chlorophyll, and Class II waters with more complex spectra contributed by a combination of constituents including chlorophyll, suspended nonalgal particulates, and dissolved organic matter [26]. More recently, using hyperspectral imagery, 22 OWT classes have been identified, including 13 for freshwater bodies and 9 for marine systems, with only some overlap between the two [27]. Fewer optical classes have been identified using multispectral imagery using techniques such as fuzzy clustering [28,29].

Many challenges are currently limiting the development of robust chlorophyll and HAB estimation using remote sensing at fine scales in estuarine systems. Traditionally, ocean color algorithms (OC2, OC3) relied on absorbance in the blue and green bands which is also influenced by suspended sediment and colored dissolved organic matter (CDOM) which are more prevalent in coastal systems [30]. In more turbid waters, researchers have had more success with chlorophyll algorithms based on ratios and differences involving red and NIR bands [19,23]. Atmospheric correction procedures can also affect the success of chlorophyll algorithms [31]. Sentinel-2 Level 2C images have been corrected for atmospheric effects using Sen2COR, but Sen2COR is optimized for corrections over land, rather than water [32], so other atmospheric corrections and cloud and cloud-shadow masking procedures are under investigation for use over water (https://ioccg.org/group/atm-corr/, accessed on 19 September 2024). Some researchers prefer to focus on spectral-shape indices which sometimes can be applied without atmospheric corrections [24], but performance without corrections has been inconsistent for estuarine systems in California [33].

Chlorophyll algorithms are less well developed and tested in estuaries due to the complex optical properties of coastal systems, and some algorithms are optimized only for specific water types. Thus, a secondary goal of our work is to characterize the optical water classes, similar to Le [34], represented in our matched database so that algorithm performance testing can be accomplished across optical water classes.

## 2. Materials and Methods

### 2.1. Water Quality Data Sources

**Figure 1.**
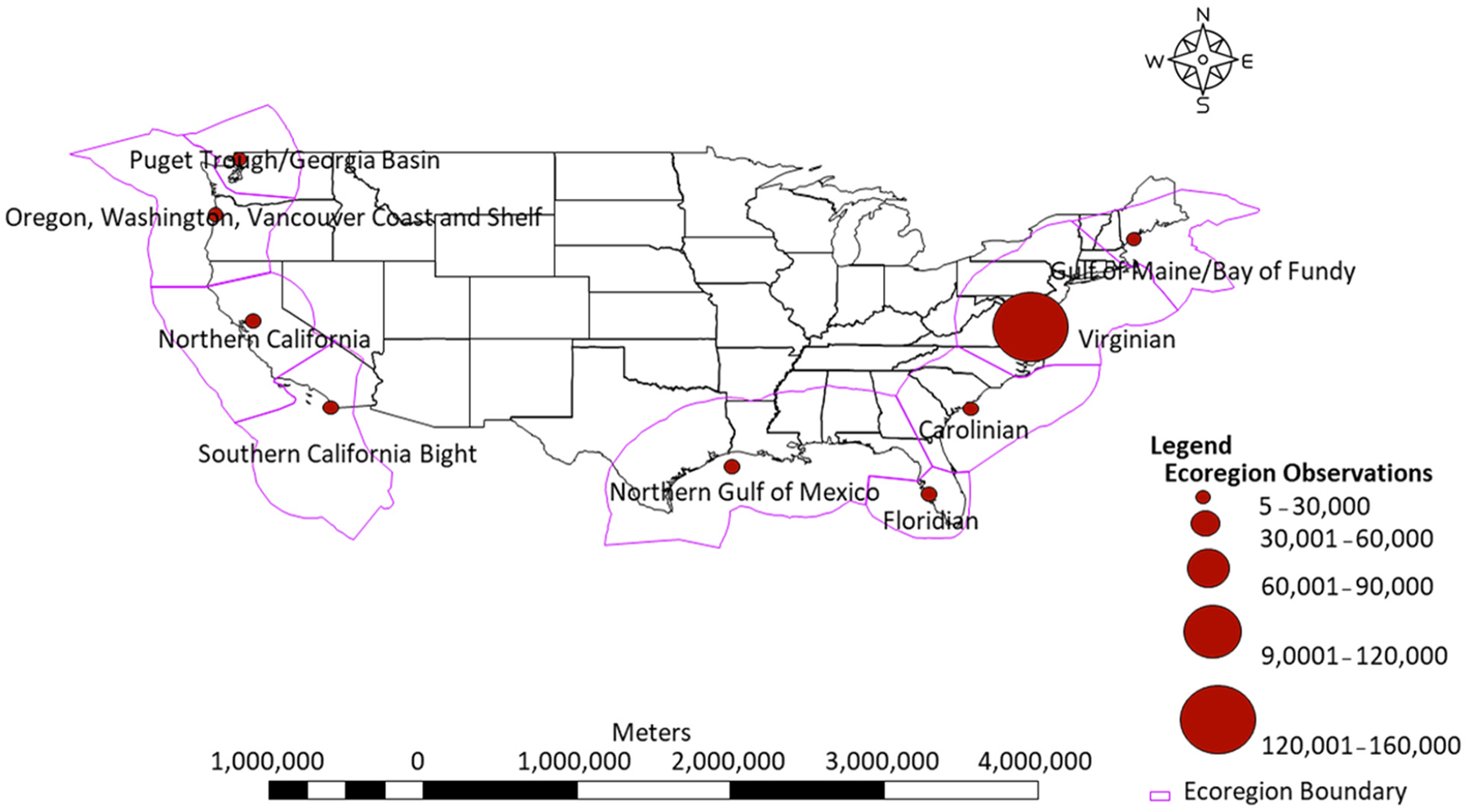
Figure depicts the distribution of sampling stations and observations by marine ecoregion in EstuarySAT. Magenta boundaries denote ecoregions, and circles represent sample observation frequencies. There are 9028 individual water quality sampling stations matched with Sentinel-2 Level 1C and 1818 water quality sampling stations matched with Sentinel Level 2A image tiles.

We assembled publicly available data sources for chlorophyll and ancillary water quality data in estuaries and freshwater tidal rivers of the coterminous United States for the period 1984–2021 using internet searches, known databases, literature searches, and personal communications (Table 1). We expanded potential data sources beyond existing databases that focus on results from grab samples (AquaSat, NWQP) to include continuous monitoring data from in situ sensors. Sources included the AquaSat database [15], the NWQP [14], USGS real-time sensor datasets, sensor data from the Northeastern Regional Association of Coastal Ocean Observing Systems (NERACOOS) regional portals, the National Estuarine Research Reserves (NERRS), state monitoring programs, place-based estuary monitoring programs (Chesapeake Bay, Long Island Sound, Narragansett Bay), and datasets associated with research publications, including those cataloged in the USGS Science Data Catalog (SDC; data.usgs.gov) (Figure 1; Table 1).

Although our immediate goal was to match chlorophyll observations with data from Sentinel-2 overpasses (available for 2015 to the present), we included a larger time range of water quality data in our retrievals to facilitate future matching with other remote sensing data as desired (e.g., Landsat) [15]. Ancillary data variables included attributes known to influence the performance of remote sensing algorithms and/or to affect the development of cyanobacteria blooms: dissolved organic carbon (DOC), turbidity, salinity, temperature, dissolved oxygen, and pH, where available.

The AquaSat database [15] combines data from Landsat 5, 7, and 8 and the National Water Quality Data Portal (NWQP) [14]. Data in AquaSat were retrieved by Ross et al. from the National Water Quality Portal [14] and the NE-LAGOS dataset [35,36] which includes harmonized lake water quality data. Most of the data in AquaSat are from freshwater systems, but some data from estuaries and freshwater tidal rivers are included.

### 2.2. Methods to Harmonize the Water Quality Datasets

We used the open-source statistical package R [37] to develop custom scripts to import data from each source and to harmonize data across sources, building upon the original code designed by Ross [15] to populate the AquaSat database. We provide a general description of dataset processing below, and more details on the processing of individual data sources can be found in our source code for data source harmonization provided in the Supplementary Material. The original AquaSat database included data retrieved from the National Water Quality Portal (NWQP) for the years 1984–May 2019. We modified the code from AquaSat to extract and format additional data for May 2019–June 2021. Other variables, including latitude/longitude, date, time, depth, and quality assurance flags were added to the table structure. Time zones were adjusted as needed to ensure the database only contained UTC (Coordinated Universal Time) units. Where time data were missing, the standard time for the collection site’s geographic location was used. We also categorized reported observation depths as surface (<3 m depth), middle (3–6 m depth), and bottom (>6 m depth).

Due to the differences in source dataset structure, we needed to harmonize variables across data sources. This processing unified data header names, variable units, sampling methods, analytical methods, and reporting of our primary variable chlorophyll collection methods. For chlorophyll, data were reported across all sources as either “Chlorophyll”, “Chlorophyll-A”, “Chl_F”, or “fluorescence”. We created a new variable “chl_category” to unify these data into reclassed categories CHL = “Chlorophyll”, CHLA = “Chlorophyll A”, CHL_Fluor = “Chl_F”, and fluorescence. Some ancillary data were reported in relative reflectance units (RFU), but those data were not preserved in the final EstuarySAT database because units are not comparable across systems.

### 2.3. Quality Assurance Processing for Water Quality Data

Ross [15] implemented several procedures in their automated retrieval process to ensure data quality, including checks to ensure that method names in the WQP were consistent with the variable name, harmonization of data units, inclusion of depth data where available, removal or consolidation of duplicate data (verification that only one observation was retained per site at a particular date and/or time), and filtering in situ data to eliminate extreme/out-of-range data: Chl-*a* > 0.01 and <10,000 μg/L; TSS > 0.01 and <100,000 mg/L; and DOC > 0.01 and <500 mg/L. Unlike Ross [15], we did not retain samples without recorded depths when matching with satellite data. We did not filter additional data retrievals based on specific thresholds but did check for anomalous data based on a review of data distributions and knowledge of reasonable values for coastal systems. In addition, we retained and reviewed quality assurance codes associated with raw data to filter out values that did not pass the originators’ quality assurance and control (QAQC) checks. QAQC flags present in the original data were retained for the user to apply further filtering as desired.

### 2.4. Sentinel-2 MSI Image Catalog Development and Pre-Processing

**Figure 2.**
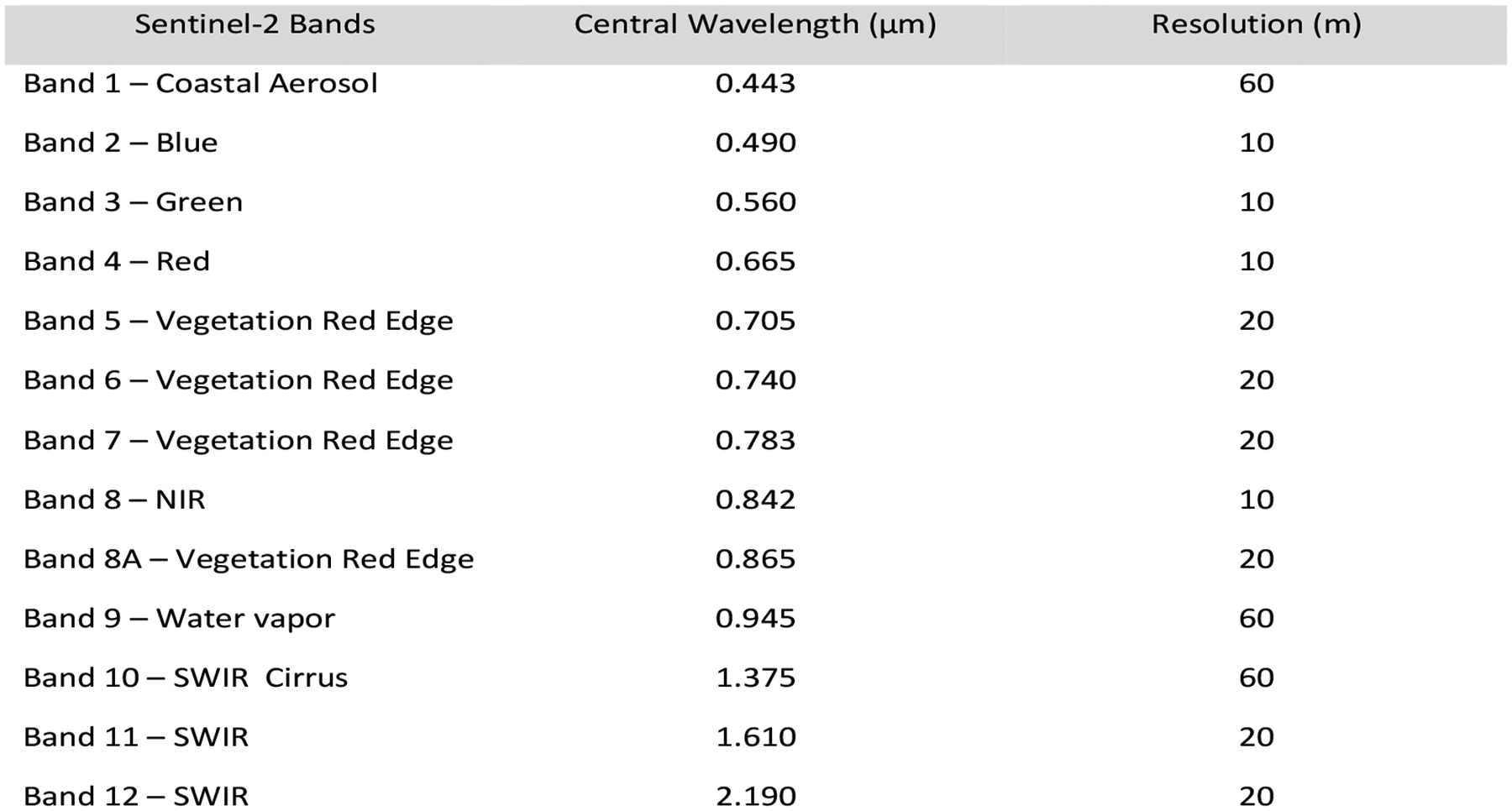
Spectral bandwidth of Sentinel-2 (A/B) with relative resolution. Source: https://www.satimagingcorp.com/satellite-sensors/other-satellite-sensors/sentinel-2a/, accessed on 19 September 2024.

The Sentinel-2 (A/B) MSI satellite platforms collect imagery of the Earth’s surface every 5–7 days. Image tiles, called granules, are fixed in size at 100 km^2^. The optical sensor system collects imagery data in the visible, near-infrared, and shortwave wavelengths. The MSI imagery comprises (13) spectral bands (Figure 2).

From both Sentinel-2 platforms, A and B, data products are provided as Level 1C and Level 2A products. The Sentinel-2A satellite was launched by the European Space Agency on 23 June 2015 and operates with a 10-day repeat cycle. The second identical satellite (Sentinel-2B) was launched on 7 March 2017. Together, they provide coverage of all Earth’s land surfaces, large islands, and inland and coastal waters every five days. Level 1C products are provided as top-of-atmosphere (TOA) and have no atmospheric correction applied to the imagery. Level 2A products are atmospherically corrected by the European Space Agency into surface reflectance imagery data, using the Sen2Cor algorithm (SNAP) [38]. Atmospheric corrections can include adjustments to reduce the influence of aerosols on reflectances and, in some cases, to remove interferences from the sun glint on the water’s surface [39]. Incorporating only the Sentinel-2 Level 2A data would have limited our water quality site to satellite imagery matches because Sentinel-2 Level 2A data were only available for the United States back to 2017, while Sentinel-2 Level 1C products were available back to 2015. To maximize the probability of finding date, time, and location matches between the water quality data and remote sensing imagery, we chose to incorporate Sentinel-2 Level 1C imagery in our matching. Incorporation of data from the Level 1C images will require atmospheric correction, which will be discussed in a subsequent paper.

### 2.5. Spatially and Temporally Matching Sentinel-2 Surface Reflectances with In Situ Water Quality Data

**Figure 3.**
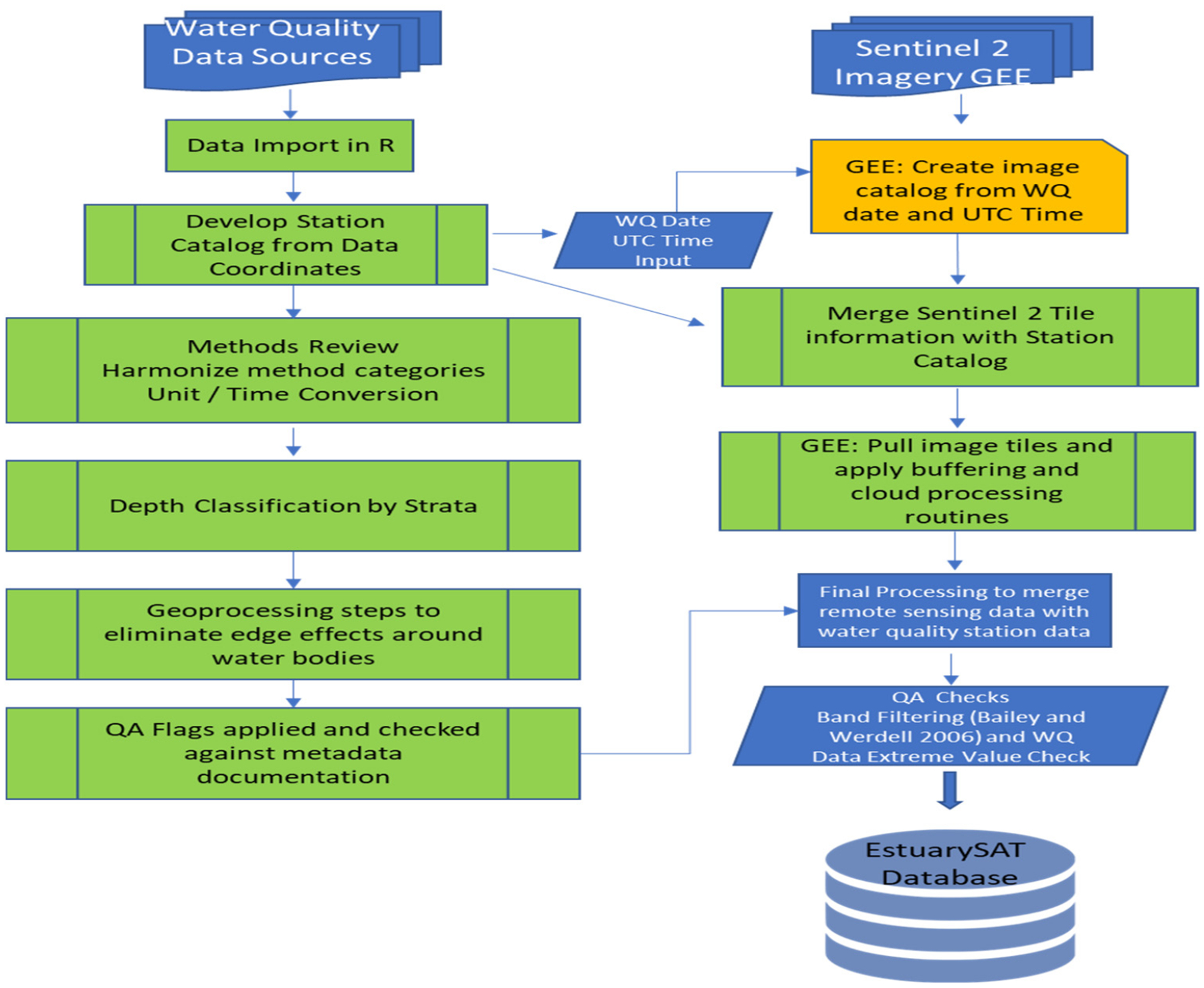
EstuarySAT database development workflow.

The first step in filtering in situ water quality data for matching with Sentinel-2 surface reflectances was to identify observation points for in situ chlorophyll data from 2015–2021 falling within estuaries and freshwater tidal rivers (see Figure 3 for workflow).

We used estuarine boundaries from the US Environmental Protection Agency’s application Estuary Data Mapper (EDM) [40] buffered by 30 m to select AquaSat, new WQP observations of interest, and other sources and to assign estuary codes (ESTCODE) from EDM. A 30 m shoreline buffer was used to allow for potential errors in recorded site locations. Only estuarine water quality observations were extracted and retrieved from AquaSat and WQP, respectively.

We used a combination of Python and R code to facilitate Sentinel-2 data matches and downloads, with the R reticulate package [41] serving as a wrapper for the Python implementation of Google Earth Engine (GEE) commands [15]. Imagery was searched and downloaded using GEE, a cloud-based geospatial platform [42,43] (see Supplementary Material). Google Earth Engine maintains an inventory of satellite imagery including Sentinel-2 and geospatial datasets with global analysis capabilities in the cloud. The estuarine chlorophyll sample points were buffered by a 30 m radius for the extraction of surface reflectance data. (Ross et al. used a 200 m radius for matches with the coarser-resolution Landsat imagery [15].) Buffered sample points that intersected shorelines or buffered roads (representing bridges or causeways) were excluded from the matching dataset to avoid remote-sensing edge effects from shadowing. Buffered estuarine sample point locations and the date/time derived from the water quality sampling stations were used to create an inventory of Sentinel-2 tiles for retrieval. We identified Sentinel-2 tiles with satellite overpass times within one day (plus or minus) of water quality sampling in Coordinated Universal Time (UTC) units.

We matched the measured water quality data to both Sentinel-2 Level 1C and Sentinel-2 Level 2A products in the GEE workspace, matching the following unique identifiers: project name, sampling station, and latitude and longitude. First, we used the “JRC_GSW1_2_Global SurfaceWater” image in GEE to check whether each sampling location falls in water pixels (https://developers.google.com/earth-engine/datasets/catalog/JRC_GSW1_2_GlobalSurfaceWater, accessed on 19 September 2024) [44]. Since the water depth within estuaries is constantly changing with the tides, some sites located near the coastal boundaries could be periodically classified as non-water regions. Thus, the surface water image is used here to add a variable for the percentage of time each extraction point falls on a pixel classified as water. We set a threshold for image pixels at 80%, meaning that the pixels within the site radius (30 m) have been classified as water at least 80% of the time.

We needed to mask remote sensing data for clouds and cloud shadows. ESA generates a flag for cloud pixels in Sentinel Level 2A imagery (Q60), but the accuracy of their cloud detection algorithms in Sen2Cor, particularly over water, has been questioned. A cloud probability mask for Sentinel-2 imagery (S2cloudless) based on an improved algorithm has been generated for the full Sentinel-2 imagery archive (https://developers.google.com/earth-engine/datasets/catalog/COPERNICUS_S2_CLOUD_PROBABILITY, accessed on 19 September 2024) We used GEE to simultaneously retrieve matching tiles with the S2cloudless cloud probabilities and to generate additional cloud shadow masks from these.

We generated a cloud shadow mask based on a conservative cloud probability threshold of 20%. GEE script developers suggest selecting a threshold based on the visual inspection of cloud probability histograms and identification of a point midway between bimodal peaks (https://medium.com/google-earth/more-accurate-and-flexible-cloud-masking-for-sentinel-2-images-766897a9ba5f, accessed on 19 September 2024). In practice, we found a second set of peaks between the low and high ends of cloud probabilities possibly linked to a specific class of clouds that varied with region and season. Thus, we chose a more conservative threshold to try to eliminate this secondary class of cloud cover. The original cloud shadow mask protocol defined shadows by cloud projection intersection with low-reflectance near-infrared (NIR) pixels. This approach would work for remote sensing over land pixels but would screen out water pixels which also have low-reflectance NIR, so we eliminated the threshold for low NIR in the process. Thus, our definition of cloud shadow masks may be overly conservative. Zenith and azimuth angles were used later to reduce matchups to images with potential sun glint issues, following the protocol of Bailey and Werdell [45].

For the final selected points, we extracted a list of pixel values for each band from 430 mm (Band 1) to 850 mm (Band 12) within a 30 m radius of observation points (aerosol, blue, green, red, NIR, red edge1-4, SWIR1-2), the median value of reflectances associated with each band, and the standard deviation of values. Information on tile-scale cloud coverage, zenith angle, azimuth angle, and water coverage percent was also retained. Final matched datasets with Sentinel-2 Level 1C and Sentinel-2 Level 2A data were filtered for quality based on protocols outlined in Bailey and Werdell [45]. Matched observations were retained if at least nine valid 10 m pixel values were available within the 30 m radius, and the median coefficient of variation among filtered reflectance values (aerosol, blue, green, red bands) was ≤0.15 after removing outliers.

### 2.6. Optical Water Classification and Clustering

It is critical for researchers to be able to evaluate the performance of remote sensing algorithms in coastal systems across a range of water types because the optical characteristics of the water column can affect calculated outputs and the accuracy of different algorithms [34]. We could not use the ancillary water quality variables (i.e., turbidity, DOC, and CDOM) in our database to describe optical water classes across all observations due to gaps in coverage, so we chose to use two methods to optically classify our systems. First, we applied a trophic state indexing method (TSI) from Bricker [46] using chlorophyll data. Second, fuzzy cluster analysis of reflectance signature profiles was applied using methods identified in Jackson [47] and Bi [29] to identify groups with similar properties. Fuzzy cluster analysis was accomplished using the cmeans function within the R package e1071 (https://www.rdocumentation.org/packages/e1071/versions/1.7-9/topics/cmeans, accessed on 19 September 2024) [48]. The optimum number of clusters was determined by evaluating eight performance indices expected to achieve maximum or minimum values at the optimum cluster number. Using the most complete water quality parameters available for our Level 2 matched dataset (chlorophyll, dissolved oxygen, turbidity, salinity, temperature), we tried to determine how these optical clusters differed in water quality. Many of the water quality parameters could not be normalized through simple transformations due to the multimodal nature of distributions. Thus, we applied a nonparametric discriminant function analysis in SAS (proc discrim method = npar) with nearest-neighbor techniques (k = 4) to evaluate which combination of water quality variables could best explain inter-cluster differences.

## 3. Results

### 3.1. Database Characteristics

The database contains water quality data from estuaries and tidal rivers suitable for matching with our remote sensing images from two Sentinel-2 products: the Sentinel-2 Level 1C data product and the Sentinel-2A product (Copernicus Sentinel data, 2015–2021). The full database structure is outlined in Table S1, with summaries of the surface-matched dataset water quality parameters described in Table 2a,b.

The database contains 26 remote sensing parameters and 10 parameters related to water quality and spatial/time referencing (Table 2, Supplementary Material). The water quality database contains *n* = 299,851 observations distributed across 9846 sampling sites, with 1818 sites matched to Sentinel-2 Level 2A images and 8028 sites matched to Sentinel-2 Level 1C images (Table 3; Figure 1). The total number of database (±24 h) matched observations after band filtering were Level 1C *n* = 84,438 and *n* = 9761 for Sentinel-2 Level 2A (Table 3). Chlorophyll observations, where available, spanned 2015–2020.

Water quality observation sites were unevenly distributed across the coastal US, with some states and estuary regions having high densities of observations, particularly on the East and West Coasts (Figure 1). However, there are data gaps on the East and West Coasts where quality and or high-frequency observation data were not available for all estuaries within our regions of interest (Figure 1). In vivo sensor-based observations make up the majority of samples, with the greatest concentration of sites and observations occurring in the Carolinian Marine Ecoregion (Figure 1). Most of our water quality data retained after quality assurance filtering span from 2015 through 2020, and Sentinel-2 imagery collected was dated 2015 through 2021. CDOM and DOC data were not retained due to reported instrument units in relative fluorescence units (RFU).

### 3.2. Chlorophyll Results

The main purpose of these datasets is the development of chlorophyll and cyanobacteria predictions for tidal and estuarine waters. Thus, chlorophyll data were prioritized during database development. Retained were samples, either in vitro or in vivo, collected within 3 m of the surface (Level 1C mean depth = 1.2 m; Level 2A mean depth = 0.64 m) (Table 2a,b). Mean chlorophyll values varied slightly between Sentinel-2 Level 1C ($\bar{x}$ = 3.3 μg/L; range 0–420 μg/L) and Level 2A ($\bar{x}$ = 7.77 μg/L; range 0.11–200.41 μg/L) (Table 2a,b). Chlorophyll samples were primarily in vivo with a percentage of total samples (90.17% from Level 1C; 9.83% from Level 2A) (Table 3). The database “methods category” break-down for Level 1C was 84,193 in vivo observations and 245 in vitro observations, while Level 2A had 9183 in vivo observations and 578 in vitro observations (Table 3). Method identification for in vivo samples was either specified by the manufacturer of the sensors or simply labeled “sensor” if more details were not available. The in vitro sample analysis generally followed US EPA Methods 445.0 and 447.0 [49,50]. Samples where the method was not defined or ambiguously labeled were not included.

**Figure 4.**
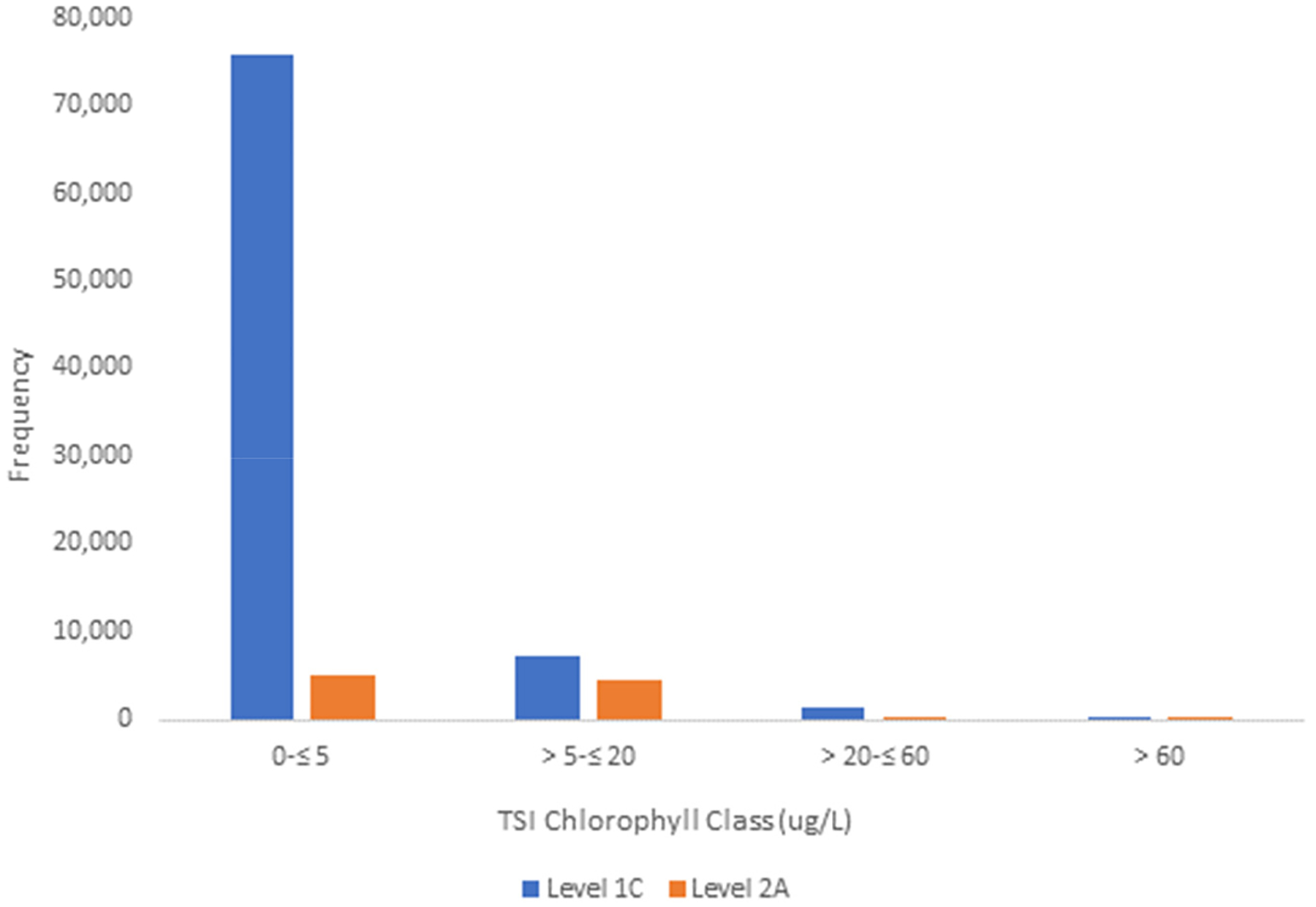
Frequency distribution of Bricker et al.’s trophic categories [31]. Trophic State Classes (Sentinel Level 1C and 2A).

Chlorophyll values spanned all four Bricker (2003) trophic state chlorophyll classes (Figure 4). The TSI distributions were similar between Sentinel-2 Level 1C and Level 2A datasets (Table 4). For Level 1C, 89.6% of observations were in Bricker class Low (0–≤5 μg/L chlorophyll) (Table 4), 8.3% were in Medium (5–≤20 μg/L chlorophyll), and 2.0% were in High and Hypereutrophic combined (>20 μg/L chlorophyll). In Level 2A, 51.2% of samples were in class Low (0–≤5 μg/L chlorophyll), 45.3% in Medium (5–≤20 μg/L chlorophyll) (Table 4), and 3.6% in High and Hypereutrophic combined (>20 μg/L chlorophyll) (Table 4). The data are strongly unimodal in both Level 1C and Level 2A observations (Figure 4).

### 3.3. Other Water Quality Parameters

Most Sentinel-2 Level 1C samples were not well oxygenated (mean = 2.8 mg DO/L; range 0–10.0 mg/L). Temperature had a mean of 8.4 C (range 0–34 C). Mean salinity was 3.8 ppt with a range of 0–36 ppt (Table 2a). Mean turbidity was 9.4 NTU (range −0.50–32.0 NTU (Table 2a). Level 1C salinity values spanned the range from freshwater (0 ppt) to normal estuarine salinities 36.0 ppt, but most samples were in the oligohaline range (Table 2a). After final processing, chlorophyll values were present for every observation, followed by temperature, dissolved oxygen, and salinity (100.0% of observations), depth (99.89%), and turbidity (99.67%) (Table 2a).

Sentinel-2 Level 2A samples appear well oxygenated (mean = 11.91 mg DO/L), with a mean temperature showing predominantly warmer water (mean = 18.65 C) (Table 2b). The lowest reported oxygen values were anoxic (min = 0.08 mg DO/L) with a maximum of 26.59 mg DO/L (Table 2b). Turbidity values were lower than Sentinel-2 Level 1C data (mean = 5.34 NTU) (Table 2b). Sentinel-2 Level 2A salinities were also mesohaline (mean = 14.73 ppt; range 0.60–35.70 ppt) (Table 2b). The final Level 2A dataset also has chlorophyll data present in 100% of observations, followed by temperature (93.41%), turbidity (93.33%), salinity (91.41%), and dissolved oxygen (90.44%) (Table 2b).

### 3.4. Fuzzy Cluster Analysis

**Figure 5.**
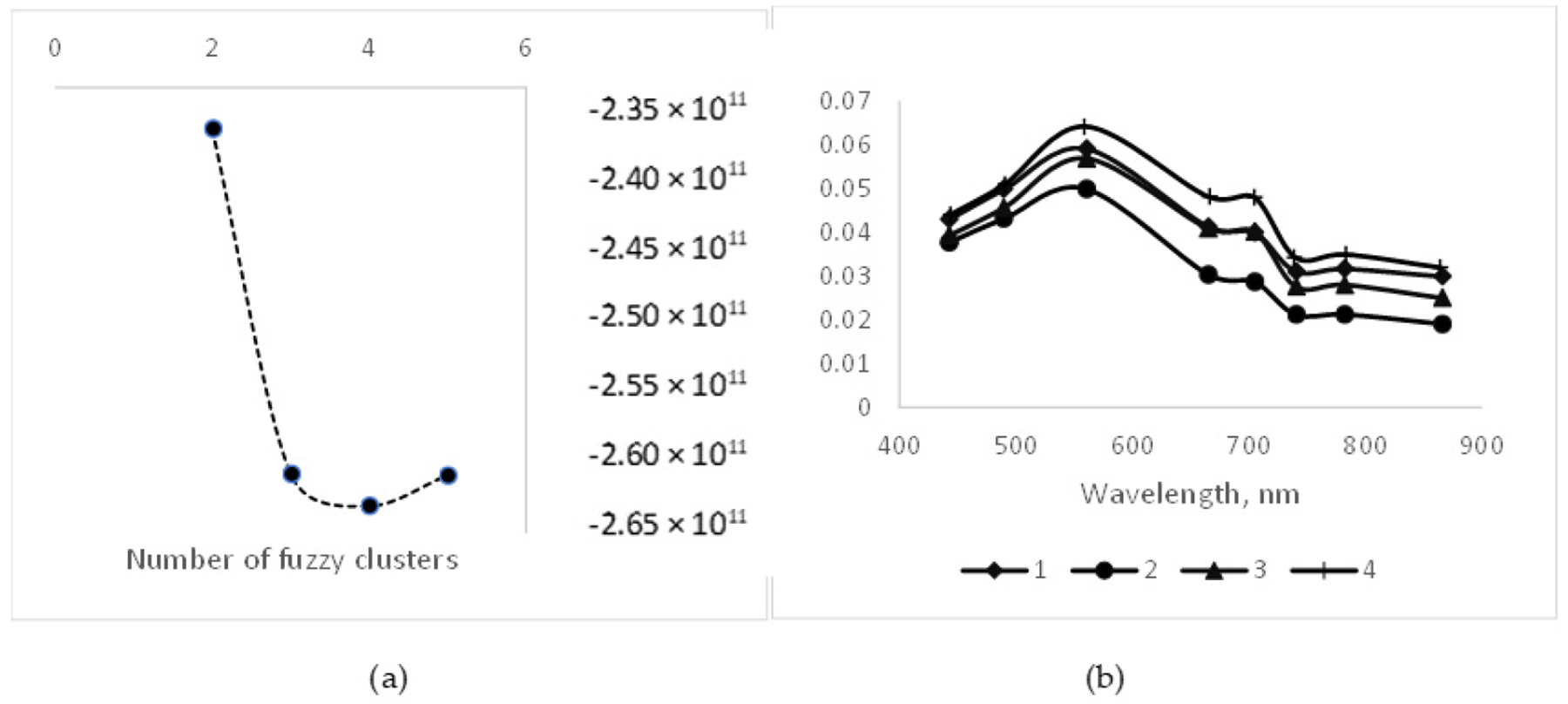
(**a**) Results from fuzzy cluster analysis (si-max) showing minimum (optimum) index value at four resolvable clusters for optical water classes. (**b**) Optical spectrum patterns for centroids of four fuzzy clusters showing reflectance peaks at 560 and 705 nm and magnitude differences across clusters.

We used fuzzy clustering to classify our Level 2A systems into optical classes using a cmeans fit indexing procedure. Fit indices calculated by the cmeans procedure yielded different decision criteria concerning the optimum number of optical water classes represented by our Sentinel-2 Level 2A matched dataset. Based on the threshold identified by the fs and apd statistics in this procedure, we chose to classify the water quality dataset into four categories (Figure 5a). The four classes follow a similar pattern, with a peak in the green region (560 nm) and a lesser secondary peak at the red edge 1 wavelength (705 nm), but vary in the overall magnitude of reflectances (Figure 5b). Similar patterns were observed in plots of center clusters based on two or six classes. The results were similar to those of previous investigators for half of their classes [43] and for Aeronet sites dominated by CDOM [51]. The second optical pattern observed by Moore [43] but not observed in our database with continuous decreases in reflectance over the full spectrum of wavelengths is typical of chlorophyll-dominated waters but with lower productivity [51]. There were no clear distinctions across optical water clusters based on trophic class (Table 5).

**Figure 6.**
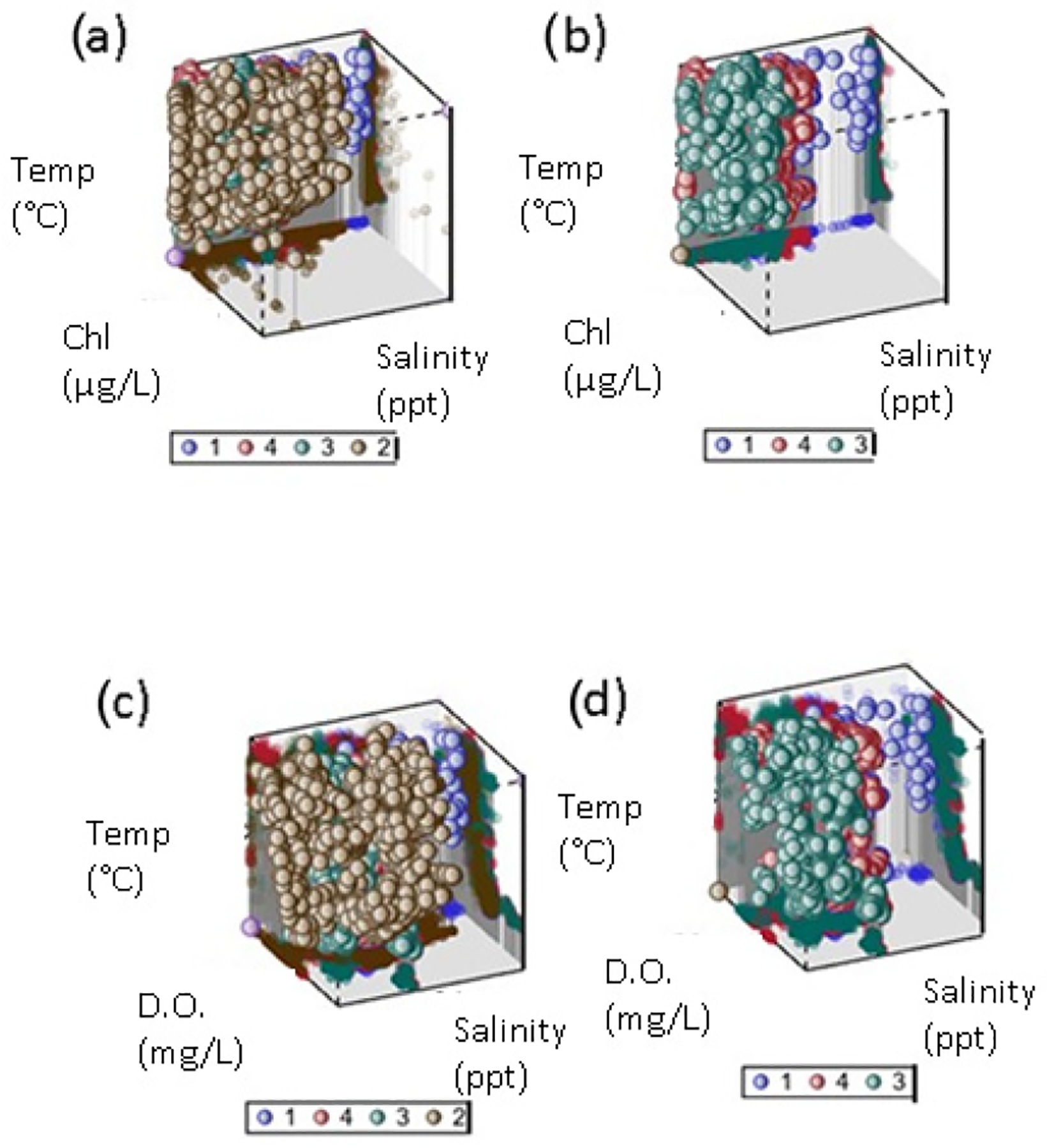
Optical clusters plotted in 3D water quality space. (**a**) Clusters 1–4 plotted as function of temperature (degrees C), chlorophyll (μg/L), and salinity (ppt). (**b**) Clusters 1–3 plotted as function of temperature (degrees C), chlorophyll (μg/L), and salinity (ppt). (**c**) Clusters 1–4 plotted as function of temperature (degrees C), dissolved oxygen (mg/L), and salinity (ppt). (**d**) Clusters 1–3 plotted as function of temperature (degrees C), dissolved oxygen (mg/L), and salinity (ppt). Clear circle represents axis origin, not a cluster.

We included all commonly available water quality parameters in a nonparametric discriminant function analysis to determine which combination of water quality parameters best explained differences among optical clusters from fuzzy cluster analysis. We found the optical clusters were well discriminated, with an overall classification error rate of about 10% (Table 6). Nonparametric DFA does not produce a simple discriminant function like a linear discriminant function so we applied a stepwise procedure to determine which combination of water quality variables (*n* = 1, 2, …, 5) produced the lowest classification error rate. Four variables, added in the order of salinity, temperature, dissolved oxygen, and chlorophyll, produced a similar overall error rate as all five together. In the previous iteration, the inclusion of salinity, temperature, and dissolved oxygen produced a similar error rate as including salinity, temperature, and chlorophyll, suggesting DO and chlorophyll may be serving as proxies for one another (Figure 6b,d). Although there is significant overlap, optical cluster 2 tends to have both higher chlorophyll and dissolved oxygen than the other three clusters (Figure 6a,c). Clusters 1, 3, and 4 appear to be separated mainly along the salinity axis (Figure 6b,d). While turbidity did not feature in the multivariate discrimination among optical clusters, it did provide a very low error rate for distinguishing cluster 4 when used alone (Table 6).

## 4. Discussion

The matched database contains 84,438 Sentinel-2 Level 1C observations and 9761 Sentinel-2 Level 2A observations matching water quality data with Sentinel-2 Level 1C and Level 2A image tiles (Table 3). As we assumed, we found more matching data when we incorporated Sentinel-2 Level 1C data than Level 2A as non-atmospherically corrected imagery is available for a longer time period from ESA, and our data quality processing excluded additional image tiles. Data and stations were assembled from sources based on found data (Table 1). These data were a mixture of publicly available data from internet sources, data derived from literature searches, and personal contacts. Our goal was to develop a database that maximized the number of water quality data observations matched with remote sensing imagery, knowing that during intermediate processing, cloud removal, and QA flagging processes, additional data would be removed from the database.

Spatially, our observation data are not evenly distributed throughout ecoregions sampled along the East, West, and Gulf Coasts (Figure 1). Our station sites are concentrated in specific estuaries and ecoregions with large monitoring programs (Figure 1). Station and observation densities vary between programs and ecoregions (Figure 1). This is likely due to the activity of water quality monitoring and estuary programs in (NEPs, NERRs) throughout the USA and is indicative of not only areas of environmental concern but also how programs deploy resources in these areas. High sample densities, not depicted in Figure 1, were seen in Long Island Sound, Chesapeake Bay, Pamlico Sound, South Coastal Florida (East and West Coasts), Apalachicola Bay, Corpus Christi Bay, San Francisco Bay, and some smaller densities in the Northwest in Oregon and Washington State. In Long Island Sound, for example, the USGS has been monitoring stream water quality for over 43 years [52]. In Chesapeake Bay, agencies and institutions began intensively monitoring the Bay in 1984. This monitoring program is a collective effort that comprises state agencies in Maryland, Pennsylvania, and Virginia and includes NGOs and research/educational institutions [53]. High-density and high-frequency monitoring program sites increase the probability of finding a match between Sentinel-2 imagery and a given site, especially with the frequent overpass schedule of Sentinel-2 satellites.

The biggest challenge in developing this database was harmonizing methods across monitoring programs. A careful review of metadata was necessary to resolve parameter name and method differences so that the field coding remained uniform throughout the processing iterations needed to generate the final product. It was also necessary to manually review some full datasets and individual files to account for changes in parameter names and formats that may have occurred throughout the monitoring programs’ lifetimes. This also required R code to be customized for specific datasets that had shifting data formats and naming conventions across years. Some other difficulties we experienced were inconsistent quality control measures and workflows within and between data sources. We discovered it was not uncommon for QA flags to change coding and or meaning throughout a program’s life cycle, particularly in some of the long-term buoy datasets. Part of the matching and harmonization process required us to make decisions regarding the handling of missing data. To a large extent, we found matching water quality observations in continuous or semi-continuous records for most of our sample sites. Some parameters such as CDOM and DOC were not well represented in the water quality time-series records. Also, the units and methods reported for CDOM and DOC were variable and sometimes not documented. Ultimately, due to missing calibration information, we determined that all CDOM records in RFU would not be included in the final database files as processing progressed. They were retained in the unfiltered intermediate database for future review.

Chlorophyll presented some particular challenges not inherent in some other supporting water quality data. Reporting units varied across data sources for chlorophyll, some reporting concentration and others only reporting raw RFU units from in situ sensor systems. The RFU data were not included in our harmonized database as we could locate no supporting calibration information relating chlorophyll sensor response (voltage) with the resultant RFU, which makes conversion to concentration units impossible. In some datasets, phaeophytin was not accounted for in the sample analysis or documentation. In those cases, chlorophyll was retained if reported as Chl-*a* (total chlorophyll). Since phaeophytin can interfere with Chl-*a* analysis, as its absorption and fluorescence peaks are in the same region as Chl-*a*, we only include Chl-*a* data reported with phaeophytin corrections [49,54].

Other chlorophyll data issues arise due to differences between sampling methods, both in vivo and in vitro. For laboratory methods, the accuracy and precision of chlorophyll values can be affected by sample collection, concentration techniques, storage protocols, choice of extraction solvent and method, bandwidth of spectrophotometers, and chlorophyll algorithm applied [55,56]. While earlier literature suggests that spectrophotometric or fluorometric analyses overestimate chlorophyll concentrations in comparison with HPLC measurements [57], others find that in vitro fluorometric and spectrophotometric measurements compare well with HPLC as long as allowance is made for chlorophyllides and allomers [58]. The accuracy of in vivo fluorometric methods can be more problematic, with the instrument deployments affected by biofouling over time and observations varying with phytoplankton composition, nutrient status, CDOM concentration, and non-photochemical (NP) quenching in high-intensity light environments [56]. The accuracy of individual sensors may also vary; Wet Labs ECO-Triplet fluorometers may produce Chl-*a* estimates 2–6 times greater than the extracted concentration using the standard factory sensor calibration [59]. Methods to correct for sensor biases, instrument drift, and NP quenching do exist but are not routinely applied [60].

The new EstuarySAT database provides chlorophyll data, including matched Sentinel-2 sets, across a broad array of estuary classes [61] and geographic regions of the conterminous United States. Given the range of methods used, both laboratory-based and in vivo sensor-based, and uneven geographic distribution, care must be taken in using the database for a broad-scale assessment of chlorophyll status and trends in estuaries of the US without a consideration of methodological differences. However, the diversity of types and optical classes represented will provide researchers with the opportunity to test the representativeness and robustness of different chlorophyll algorithms developed with more limited calibration datasets and using different atmospheric correction processes. Some chlorophyll algorithms such as spectral-shape indices might be used with the top-of-atmosphere reflectances available from Sentinel-2 Level 1 or with the atmospheric corrections from Sen2COR available in Sentinel-2 Level 2C datasets, but previous researchers have had mixed success [24,33], so more testing is needed. Other ratio-based algorithms will require testing in conjunction with various atmospheric correction procedures beyond the Sen2COR corrections available in Sentinel-2 Level 2C products [23].

Our matched dataset includes samples from a broad range of water quality conditions for testing purposes (clear to turbid, trophic classes 1–4, and oligosaline to hypersaline). Optical water classes described by fuzzy cluster analysis of our database had similar spectral signatures to those described as typical of CDOM-dominated waters but lacked classes characteristic of chlorophyll-dominated waters without optical interferences [45]. Although overlapping, the water quality of three of the classes appeared to be distinguished from others mainly based on salinity differences, which is often not considered in developing empirical chlorophyll algorithms (Figure 6a,d). Salinity often covaries with CDOM along an estuarine gradient, so these differences may reflect that cross-correlation. In addition, backscattering coefficients vary with both temperature and salinity, so it is reasonable to detect differences in optical signatures for oligohaline vs. mesohaline samples [62]. The EstuarySAT database can help meet the challenge of determining the robustness of existing algorithm performance across the salinity and chlorophyll gradients.

The EstuarySAT database provides the opportunity for more robust testing of existing chlorophyll algorithms within estuaries and freshwater tidal rivers at the fine spatial and temporal resolutions available from Sentinel-2. Some of these algorithms are more sensitive to the detection of cyanobacteria blooms [24,63,64], while others are not. Even in cases where algorithms can only be used to better quantify chlorophyll levels in general, it is possible to apply chlorophyll thresholds (10 and 24 μg/L) as warning indicators of potential HAB blooms that warrant targeted testing [20]. Broad-scale mapping of chlorophyll across estuaries can help in overall assessments of estuarine productivity and potential driving variables for bloom patterns in space and time. In addition, chlorophyll levels from remote sensing can be used in conjunction with other environmental variables and/or hydrobiogeochemical models to predict the likelihood of the occurrence of HABs [22,65]. The EstuarySAT database contains data from several estuarine systems with histories of HAB formation: e.g., James River, Puget Sound, Albemarle/Pamlico Sounds, San Francisco Bay St. Johns, St. Lucie, and Caloosahatchee.

In the future, we will be improving and updating the database as new water quality and improved remote sensing imagery become available. For example, the imagery provided in the GEE catalog is Sentinel Level 2A which is atmospherically corrected using ESA’s SNAP toolbox (Sen2COR correction algorithm). The literature has suggested that Sen2COR may not perform well over water [46,47]. This may affect the resultant estimated chlorophyll concentrations from the imagery data. We are currently reviewing different methods and software tools to atmospherically correct Sentinel-2 Level 1C imagery to improve any derived chlorophyll data and algorithms. Future research will include applying and examining the robustness of existing algorithms for chlorophyll and cyanobacteria bloom estimation in estuaries and tidal rivers. In the future, after adding atmospherically corrected Sentinel-2 Level 1C data, our surface site observation matching will increase by approximately 90%, with some losses after quality control, cloud, and band QA filters are applied. Our research will continue with database updates and additional observations as they become available, evaluating existing chlorophyll and cyanobacteria algorithms for prediction and examining water quality time series to predict blooms.

## Supplementary Material

Supplement1

## Figures and Tables

**Table 1.** Data sources used to develop EstuarySAT. Listed are the major geographic area, ecoregion, data site description, providing organization or agency, and link to the online data source.

Geographic Area	Ecoregion	Description	Agency	Online Source
Florida	Floridian	Florida Department of Environmental Protection Freshwater Algal Bloom Monitoring Program	Florida Department of Environmental Protection	https://floridadep.gov/dear/algal-bloom/content/algal-bloom-sampling-results, accessed on 28 October 2020
Florida	Floridian	Florida Coastal Everglades Data	Florida Coastal Everglades (LTER)	https://fcelter.fiu.edu/, accessed on 7 August 2021
Florida	Floridian	Hurricane Harvey Impacts on Sediment Biogeochemistry	Biological and Chemical Oceanography Data Management Office	https://www.bco-dmo.org/dataset/839436, accessed on 8 August 2021
Connecticut	Virginian	Buoy data from monitoring operations in Mystic River, CT	U.S. Environmental Protection Agency	https://www.epa.gov/mysticriver/basic-information-about-mystic-river-buoy, accessed on 3 July 2021
California	Northern California	Real-time water quality monitoring stations throughout California	Department of Water Resources	https://cdec.water.ca.gov/, accessed on 2 April 2021
California	Northern California	Water Quality—San Francisco Bay Project	US Geological Survey (USGS)	https://sfbay.wr.usgs.gov/water-quality-database/, accessed on 8 August 2021
Chesapeake Bay	Virginian	Chesapeake Bay Water Quality Monitoring Program	Chesapeake Bay Program	https://www.chesapeakebay.net/what/downloads/cbp-water-quality-database-1984-present, accessed on 4 July 2021
Long Island Sound	Virginian	LISICOS—The Long Island Sound Integrated Coastal Observing System	Connecticut DEEP	https:/Lisicos.uconn.edu/data_stn.php, accessed on 3 April 2021
Maryland	Virginian	Maryland Eyes on the Bay	Maryland Department of Natural Resources	http://eyesonthebay.dnr.maryland.gov/, accessed on 4 July 2021
Massachusetts	Gulf of Maine/Bay of Fundy	Buoy data from the Charles River	Massachusetts Water Resources Authority	https://www.mwra.com/search/media?s=Charles+River, accessed on 3 July 2021; https://www.epa.gov/charlesriver/live-water-quality-data-lower-charles-river, accessed on 3 July 2021
New York	Virginian	Hudson River Environmental Conditions Observing System	HRECOS & Partners	https://hrecos.org, accessed on 10 February 2021
Puget Sound	Oregon, Washington, Vancouver	Center for Coastal Margin Observation and Prediction	CMOP & Partners	http://www.stccmop.org/, accessed on 29 October 2020
Rhode Island	Virginian	Narragansett Bay Fixed Site Monitoring Network	Rhode Island Department of Environmental Management and Partners	http://www.dem.ri.gov/programs/emergencyresponse/bart/stations.php, accessed on 16 December 2020
Texas	Northern Gulf of Mexico	Hurricane Harvey Texas Lagoon data	Biological and Chemical Oceanography Data Management Office	https://www.bco-dmo.org/deployment/805271, accessed on 8 August 2021
Texas	Northern Gulf of Mexico	Hurricane Harvey Texas Lagoon data	Biological and Chemical Oceanography Data Management Office	https://www.bco-dmo.org/deployment/805239, accessed on 8 August 2021
Continental US	various	U.S. Geological Survey Water Data for the Nation	USGS	https://waterdata.usgs.gov, accessed on4 February 2021
Continental US	various	AquaSat—Paired water quality and remote sensing data	M. Ross—Colorado State University	https://github.com/GlobalHydrologyLab/AquaSat, accessed on 2 June 2021
New England Region	Virginian	Regional Ocean Observing System	NERACOOS & Partners	https://neracoos.org, accessed on 29 October 2020
US Waters	various	Historical/Real-time data from water quality buoy stations	US Environmental Protection Agency	http://www.epa.gov, accessed on 4 February 2021
US Waters	various	USGS datasets and reports for various sites throughout the US	US Geological Survey (USGS)	https://usgs.data.gov, accessed on 11 February 2021
Coastal US	various	National Estuarine Reserve System	NOAA	https://cdmo.baruch.sc.edu/get/landing.cfm, accessed on 15 April 2021
Continental US	various	USGS Data Science for Water Resources	U.S. Geological Survey	https://www.usgs.gov/mission-areas/water-resources, accessed on 11 February 2021
South Coastal US	various	Southeast Coastal Ocean Observing Regional Association (SECOORA)	SECOORA	https://portal.secoora.org, accessed on 14 December 2020

**Table 2.** (**a**) Surface summary statistics for water quality parameters matched with Sentinel Level 1C (24 h data averages). (**b**) Surface summary statistics for water quality parameters matched with Sentinel Level 2A (24 h averages).

(a)						
Parameter	N	Mean	Std. Dev.	Min	Max	Percent Obs.
Depth of sample (m)	84,344	1.20	0.52	0.13	3.00	99.89%
Temperature (C)	84,438	8.40	12.00	0.00	34.00	100.00%
Dissolved oxygen (mg/L)	84,438	2.80	3.80	0.00	10.00	100.00%
Salinity (ppt)	84,438	3.80	6.10	0.00	36.00	100.00%
Turbidity (NTU)	84,160	9.40	14.00	−0.50	332.00	99.67%
Chlorophyll (μg/L)	84,438	3.30	13.00	0.00	420.40	100.00%
Total observations	84,438					
(b)						
Parameter	N	Mean	Std. Dev.	Min	Max	Percent Obs.
Depth of sample (m)	9761	0.64	0.46	0.10	3.00	100.00%
Temperature (C)	9118	18.65	8.99	3.81	33.62	93.41%
Dissolved oxygen (mg/L)	8828	11.91	3.61	0.08	26.59	90.44%
Salinity (ppt)	8923	14.73	5.99	0.06	35.70	91.41%
Turbidity (NTU)	9110	5.34	9.58	0.00	147.68	93.33%
Chlorophyll (μg/L)	9761	7.77	7.15	0.11	200.41	100.00%
Total observations	9761					

**Table 3.** Database characteristics and observation counts.

Processing Level	Total Observations	Level 1C	Level 2A	Level 1C Percent Total (In Vivo/In Vitro)	Level 2A Percent Total (In Vivo/In Vitro)
All Observations	299,851	252,536	47,315	84.22%	15.78%
Matched	94,199	84,438	9761		
Matched Chlorophyll					
In Vivo	93,376	84,193	9183	90.17%	9.83%
In Vitro	823	245	578	29.77%	70.23%

**Table 4.** Bricker et al.’s Trophic State Class Frequencies. The table depicts a trophic state index categorization using chlorophyll data based on classes suggested by Bricker [46].

TSIc	Chlorophyll Range (μg/L)	Level 1C Frequency	Level 2A Frequency	Percent Total Level 1C	Percent Total Level 2A
Low	0–≤5	75,690	4998	89.6%	51.2%
Medium	>5–≤20	7044	4418	8.3%	44.4%
High	>20–≤60	1301	329	1.5%	3.3%
Hypereutrophic	>60	403	16	0.5%	0.2%
Totals		84,438	9761		

**Table 5.** Water quality statistics of optical water clusters.

Parameter	Cluster 1	Cluster 2	Cluster 3	Cluster 4
Mean Temperature (°C)	17.7	16.7	20.4	19.7
Range	3.8–31.7	3.2–32.9	4.3–32.5	4.0–33.6
Interquartile	7.8–26.7	7.7–25.1	15.4–27.5	9.2–27.7
Mean Dissolved Oxygen (mg/L)	12.4	11.6	10.9	12.0
Range	3.6–20	0.5–18.2	0.1–26.6	4.8–26.6
Interquartile	10.0–14.7	9.3–14.2	7.8–14.2	9.4–14.8
Mean Salinity (ppt)	17.5	18.7	11.6	12.2
Range	7.8–35.7	0.1–32.1	0.1–20.3	0.1–23.4
Interquartile	14.3–20.1	15.1–23.1	8.7–14.5	7.2–16.9
Mean Turbidity (NTU)	2.6	5.5	14.7	2.4
Range	0–17.5	0–112.6	2.4–158	2.4–36.6
Interquartile	2.4–2.4	2.4–2.4	2.4–36.6	2.4–2.4
Mean Chlorophyll a (μg/L)	7.2	8.6	6.0	9.8
Range	0.1–119	0.5–200.4	0.1–56.1	0.4–49.3
Interquartile	3.5–9.4	4.6–9.6	3.1–7.7	5.2–12.8

**Table 6.** Error rates for nonparametric discriminant function analysis (SAS output) with nearest-neighborhood method (k = 4) and varying number of water quality parameters included as predictors.

Cluster	1	2	3	4	Total
Parameters included					
All	0.1276	0.0828	0.0608	0.1421	**0.1036**
Chlorophyll	0.6612	0.6711	0.4798	0.5204	0.5805
Dissolved oxygen	0.7376	0.5584	0.5037	0.4322	0.5443
Salinity	**0.4673**	**0.4746**	**0.3238**	0.5371	**0.4488**
Temperature	0.7168	0.5179	0.5325	0.5376	0.567
Turbidity	0.9843	0.9429	0.6615	**0.0002**	0.6057
Salinity + Chlorophyll	0.4118	0.3587	0.263	0.3599	0.3432
Dissolved oxygen	0.3065	0.256	0.176	0.2852	0.2544
Temperature	**0.2234**	**0.1956**	**0.0968**	**0.2401**	**0.1863**
Turbidity	0.4419	0.4162	0.3121	0.4118	0.3913
Salinity + Temperature + Chlorophyll	**0.1718**	0.1203	0.085	0.1843	0.1388
Dissolved oxygen	0.1782	**0.1096**	**0.0612**	**0.1725**	**0.1296**
Turbidity	0.2153	0.1749	0.0862	0.2126	0.1691
Salinity + Temperature + Dissolved oxygen + Chlorophyll	**0.128**	**0.083**	0.0632	**0.1451**	**0.1051**
Turbidity	0.1764	0.1033	**0.0593**	0.162	0.1242

Note: Lowest overall error rate for a given number of parameters or for individual clusters within a set are highlighted in bold.

## Data Availability

The data presented in this study are openly available in the Data.gov repository at https://doi.org/10.23719/1530288.
