# Supplementary material for: EstuarySAT Database Development of Harmonized Remote Sensing and Water Quality Data for Tidal and Estuarine Systems
Source: Water (Basel). Author manuscript; Available in PMC 2025 Sep 25. (PMC11534012; doi:10.3390/w16192721)
Supplement: Supplement1 [file NIHMS2025764-supplement-Supplement1.zip › Supplemental Code File List and Description.pdf]

Supplemental Materials Code File Names and Descriptions

The table below lists the code files used in the dataset generation and matching procedure. Steps are organized 0-3 indicating grouping order of processing steps. Each file name has a brief description designating the main procedure of that step. We also include a “Type” to denote what stage the processing procedure represents in generating the final dataset.

**Step**	**File Name**	**Description**	**Type**
0	0_Aquasat_update V12622.Rmd	This code is an updated version of the original AquaSat code from Ross. It will add the most recent data from Aquasat (1984-2019) to include May 2019 through Jan 2021.	Pre-processing
0	0_Aquasat_update_addtionalvariable V12622.Rmd	Download additional variables. This code will download and process additional WQ variables; water temperature, salinity, pH and DO. Data queries only include sites within defined estuary boundaries.	Pre-processing
0	0_Aquasat_combined_wqp V12622.Rmd	This code harmonizes the updated Aquasat base raw data with the updated additional variables from *additionalvariable	Pre-processing
0	0_Aquasat_wqp_data_harmonizing V12622.Rmd	This code combines and harmonizes WQP and Aquasat updated data.	Pre-processing
0	0_ChlaBuoys V12622.Rmd	This code organizes and unifies chlorophyll buoy data from multiple sources.	Pre-processing
0	0_Other EDITED V12622.Rmd	This code is used to process other datasets.	Pre-processing
1	1_site_inventory2 V12722.Rmd	This code generates a list of sites within pre-generated estuarine boundaries and assigns an estuary code to each record and the MGRS information from Sentinel 2 image tiles	Main Processing
2	2_data_harmonized3 V12722.Rmd	This code combines and harmonizes all water quality data into one dataset.	Main Processing
3	3_Sentinel_Matching V13122.Rmd	This code produces the final matched dataset between water quality observations and Sentinel 2 imagery	Final Processing
