# Supplementary material for: EstuarySAT Database Development of Harmonized Remote Sensing and Water Quality Data for Tidal and Estuarine Systems
Source: Water (Basel). Author manuscript; Available in PMC 2025 Sep 25. (PMC11534012; doi:10.3390/w16192721)
Supplement: Supplement1 [file NIHMS2025764-supplement-Supplement1.zip › water 3190795 Supplementary Table S1.pdf]

| Variable Name | Description |
|---|---|
| ESTCODE_N | Estuary CODE with segment |
| ESTCODE | Estuary CODE |
| ESTUARY | Estuary name |
| Segment | Subestuary code |
| STATE | State Location of the Estuary |
| Ecoregion | MEOW Ecoregion (Spaulding 2007) of the Estuary |
| date_unity | Date and Time in UTC time zone |
| Time | Time information associated with the Sentinel product |
| timediff | time difference between Time and date_unity |
| tz | time zone information (UTC, Local_tz, No_tz) |
| Date | Date in UTC |
| Date_UTC | Date and Time in UTC time zone |
| SiteID | Station ID |
| .geo | Georeferencing information, including layer type and the coordinate system |
| LAT_pt | Latitude of the site point |
| LNG_pt | Longitude of the site point |
| Project | Project information to indicate the sub-dataset source |
| MGRS | Sentinel Military Grid Reference System (MGRS) tile ID |
| Sentinel_id | The Sentinel Product ID in Google Earth Engine (GEE) |
| Aerosol | Aerosol band (Band 1, 443nm, 60m resolution) |
| Aerosol_sd | Aerosol band standard deviation |
| Blue | Blue band (Band 2, 490nm, 10m resolution) |
| Blue_sd | Blue band standard deviation |
| Green | Green band (Band 3, 560nm, 10m resolution) |
| Green_sd | Green band standard deviation |
| Red | Red band (Band 4, 665nm, 10m resolution) |
| Red_sd | Red band standard deviation |
| RedEdge1 | Vegetation Red Edge 1 band (Band 5-, 705nm, 20m resolution) |
| RedEdge1_sd | Vegetation Red Edge 1 band standard deviation |
| RedEdge2 | Vegetation Red Edge 2 band (Band 6,-, 740nm, 20m resolution) |
| RedEdge2_sd | Vegetation Red Edge 2 standard deviation |
| RedEdge3 | Vegetation Red Edge 3 band (Band 7-, 783nm, 20m resolution) |
| RedEdge3_sd | Vegetation Red Edge3 standard deviation |
| NIR | Near-infrared Band 8, 842nm, 10m resolution |
| NIR_sd | NIR band standard deviation |
| RedEdge4 | Vegetation Red Edge 4 band (Band 8A-, 865nm, 20m resolution) |
| RedEdge4_sd | Vegetation Red Edge 4 standard deviation |
| SWIR1 | Short wavelength infrared 1 (Band 11, 1610nm, 20m resolution) |
| SWIR1_sd | Short wavelength infrared 1 standard deviation |

| | |
|---|---|
| SWIR2 | Short wavelength infrared 2 (Band 12, 2190nm, 20m resolution) |
| SWIR2_sd | Short wavelength infrared 2 standard deviation |
| pixelCount | the number of the pixels within the buffer region |
| Cloud | Cloud percentage from the "cloudpull.py" code. |
| Depth_m | Monitoring data depth, meters |
| Temp_C | Water temperature, degrees Centigrade |
| DO_mg.L | Dissolved oxygen, mg/L |
| Salinity_ppt | Salinity, ppt |
| Turb_NTU | Turbidity, Nephelometric Turbidity Units |
| Chl_ug.L | Chlorophyll, ug/L |
| Method | Method to measure the Chlorophyll |
| Chl_category | Chlorophyll Category: CHL, CHLA, CHLF (fluorescence-based) |
| Layer | Measurement layer (surface/middle/bottom |
| Calculated | Indicates if the flushing time and stratification is calculated for this estuary |
| GRID1MIL | Military Grid Reference System (MGRS) 1 to 1,000,000 |
| GRID100K | Military Grid Reference System (MGRS) 1 to 100,000 |
| LONGITUDE | MGRS Longitude as decimal degrees |
| LATITUDE | MGRS Latitude as decimal degrees |
| EPSG | European Petroleum Survey Group coordinate system information |
| pwater_max | Max water classification percentage |
| pwater_med | median water classification percentage |

Table S1: Database parameter list. This list identifies both the table variable and the brief description of each parameter in EstuarySAT.
